# Increased Risk of Ischemic Stroke in Patients with Benign Paroxysmal Positional Vertigo: A 9-Year Follow-Up Nationwide Population Study in Taiwan

**DOI:** 10.3389/fnagi.2014.00108

**Published:** 2014-06-02

**Authors:** Chung-Lan Kao, Yuan-Yang Cheng, Hsin-Bang Leu, Tzeng-Ji Chen, Hsin-I Ma, Jaw-Wen Chen, Shing-Jong Lin, Rai-Chi Chan

**Affiliations:** ^1^Department of Physical Medicine and Rehabilitation, Taipei Veterans General Hospital, Taipei, Taiwan; ^2^School of Medicine, National Yang-Ming University, Taipei, Taiwan; ^3^Institute of Physical Therapy and Assistive Technology, National Yang-Ming University, Taipei, Taiwan; ^4^Department of Physical Medicine and Rehabilitation, Taichung Veterans General Hospital, Taichung, Taiwan; ^5^Institute of Clinical Medicine, National Yang-Ming University, Taipei, Taiwan; ^6^Healthcare and Management Center, Taipei Veterans General Hospital, Taipei, Taiwan; ^7^Division of Cardiology, Department of Medicine, Taipei Veterans General Hospital, Taipei, Taiwan; ^8^Department of Family Medicine, Taipei Veterans General Hospital, Taipei, Taiwan; ^9^Institute of Hospital and Health Care Administration, National Yang-Ming University, Taipei, Taiwan; ^10^Department of Neurological Surgery, Tri-Service General Hospital, National Defense Medical Center, Taipei, Taiwan; ^11^Department of Medical Research and Education, Taipei Veterans General Hospital, Taipei, Taiwan; ^12^Cardiovascular Research Center, National Yang-Ming University, Taipei, Taiwan; ^13^Institute of Pharmacology, National Yang-Ming University, Taipei, Taiwan

**Keywords:** stroke, benign paroxysmal positional vertigo, dizziness, vertigo, risk factors

## Abstract

Benign paroxysmal positional vertigo (BPPV) is a common form of vertigo and is characterized by episodic dizziness related to changes in head position relative to gravity. BPPV symptoms can be similar to those of central nervous system vascular diseases. The association between BPPV and ischemic stroke has not yet been investigated. The study cohort consisted of patients who were diagnosed with BPPV at least twice in the previous year as an outpatient or for whom BPPV was the primary diagnosis as an inpatient (*n* = 4104). An age- and gender-matched sample that excluded patients with a diagnosis of any form of vertigo was selected as the comparison cohort (*n* = 8397). All cases were followed up from January 1, 2000, to December 31, 2008. The demographic characteristics, medical comorbidities, and use of medications in both groups were investigated using chi-square tests. A stratified analysis of stroke risk factors was performed to determine the hazard ratios of BPPV. During the 9-year follow-up period, 185 of the 4104 (4.5%) subjects with BPPV and 240 of the 8379 (2.9%) subjects without BPPV developed ischemic strokes. The crude hazard ratio of BPPV for developing ischemic strokes was 1.708. After adjusting for stroke risk factors, the risk of developing ischemic strokes in BPPV subjects was 1.415-fold higher than the risk among those without BPPV (confidence interval: 1.162–1.732, *p* = 0.001). After a subgroup analysis stratified according to stroke risk factors, BPPV remained independently associated with a higher risk of developing future ischemic stroke. We conclude that BPPV is independently associated with a risk of subsequent ischemic stroke. More aggressive control of modifiable risk factors for ischemic strokes should be conducted in patients with BPPV.

## Introduction

Benign paroxysmal positional vertigo (BPPV), first described by Barany, is characterized by brief episodes of vertiginous attacks precipitated by changes in head positions relative to gravity (Bárány, 1920). BPPV is one of the most common types of vertigo and causes balance disorders in the aging population (Baloh, 1992). Abnormal stimulation of the cupula by calcium carbonate debris within the semicircular canals has been proposed to cause BPPV (Schuknecht, 1969; Hall et al., 1979). The diagnosis of the canals involved in BPPV is based on the symptoms and directions of nystagmus in the Hallpike–Dix test (Dix and Hallpike, 1952; Furman and Cass, 1999).

The actual etiology of BPPV remains unclear. Advanced age, head trauma, inactivity, viral labyrinthitis, and ischemia of the anterior vestibular artery may predispose individuals to BPPV (Baloh et al., 1987; Steenerson et al., 2005). Numerous investigations have reported increased incidence in females and the aged population (Lynn et al., 1995; Angeli et al., 2003; Yimtae et al., 2003; Steenerson et al., 2005; Kao et al., 2009). Physical exercise, such as the canalith repositioning maneuver, yields a 67–94% rate of successful remission (Susan and Herdman, 2007). Physical therapy is the primary treatment after a BPPV diagnosis. Although benign in nature, BPPV can be devastating during spells of attacks, causing physical and psychological distress. Comorbid conditions, such as anxiety, depression, and chronic dizziness, may be underestimated, especially in female (Ferrari et al., 2014).

There are positional vertigo, which mimic BPPV, those include migraine, tumor or stroke, cerebellar or brainstem disorders, perilymphatic fistula, superior canal dehiscence, hypermobile stapes (Susan and Herdman, 2007). Some vestibular ischemic strokes may be perceived as inner ear dysfunctions, such as hearing loss or vertigo (Kim and Lee, 2009). In a previous study, 10.4% of patients with cerebellar infarction only showed symptoms of vestibular neuritis (Lee et al., 2006). Positional vertigo with central causes is diagnosed in 12% of patients with positional vertigo (Bertholon et al., 2006). Although central nervous lesions, such as ischemic stroke, rarely present with recurrent or positional vertigo without other neurological abnormalities (Watson et al., 1981; Estol et al., 1996), BPPV patients in the clinic often enquire if they are experiencing a stroke or if this condition could cause strokes later in life.

The risk and occurrence of ischemic stroke in BPPV patients have not been previously investigated. Therefore, this study aimed to determine the risk of subsequent ischemic stroke in patients with BPPV using a population-based study. This retrospective cohort study may provide valuable information regarding demographic risk factors and comorbidities of BPPV and its association with ischemic stroke in Taiwanese populations.

## Materials and Methods

### Database

The Taiwan National Health Research Institute (NHRI) database was used for this study. This database contains original medical data from one million individuals specifically selected by Taiwan’s National Health Insurance (NHI). The NHI is the single largest medical health insurance company in Taiwan and contains the medical registry of all board-certified physicians and contracted medical facilities. More than 98% of the Taiwanese population is included in this program. The data from the NHI program are representative of the general medical health status of the Taiwanese population. According to Taiwan NHRI, there are no significant differences in age, gender, or health care costs between the sampled patients in the database and all the beneficiaries of the NHI program. Thus, this database is a valuable source for investigating the relationship between the incidence of BPPV and ischemic stroke in Taiwan.

### Study design

We conducted a retrospective cohort study. Patients greater than 20 years of age with a BPPV diagnosis (ICD-9-CM code 386.11) from January 1, 2000 to December 31, 2008 were selected as the study cohort. To ensure a correct diagnosis, the patients must have been diagnosed with BPPV at least twice in the same year according to outpatient clinical records or have BPPV as the primary diagnosis as an inpatient. Patients with a BPPV diagnosis prior to 2000 were excluded to ensure that only new-onset cases were examined. Patients with a diagnosis of ischemic stroke prior to the BPPV diagnosis were also excluded to prevent erroneous causal relationships. A total of 4104 patients with BPPV were randomly selected as the study cohort.

Cases in the comparison cohort were randomly selected from the remaining patients in the database, and their age and gender were matched with the patients in the study cohort in order to investigate the relations between BPPV and subsequent ischemic stroke events. Patients diagnosed with any form of vertigo (ICD-9-CM code 386) were excluded to prevent the inclusion of cases of BPPV that were misdiagnosed. Patients diagnosed with ischemic stroke prior to inclusion were also excluded. A total of 8379 patients were selected as the comparison cohort. In order to evaluate the risk factors of ischemic stroke in elderly people, individuals aged more than 65 years were extracted from the cohorts for further analysis (*n* = 4389).

All the cases were followed up until December 31, 2009 to track the incidence of ischemic stroke, which was identified by insurance claims. To avoid misdiagnoses, we only enrolled patients with a primary diagnosis of ischemic stroke (ICD-9-CM code 433–436) as an inpatient. Potential outcome confounders, including age, gender, comorbidities [such as hypertension (ICD-9-CM code 401–405), diabetes mellitus (ICD-9-CM code 250), atrial fibrillation/flutter (ICD-9-CM code 427.31, 427.32), coronary artery disease (ICD-9-CM code 410–414), hyperlipidemia (ICD-9-CM code 272)], and medications (such as antiplatelets, anticoagulants, and statins) were considered. Medical comorbidities were defined as a minimum of two incidences of diagnosis as an outpatient or a primary diagnosis as an inpatient. Medications prescribed to the patients at any time during the follow-up period were identified.

### Statistical analysis

Microsoft SQL Server 2005 (Microsoft Corporation) and SPSS software (SPSS, Inc.) were used for data management and computing. A Student’s *t*-test and chi-square tests were conducted to compare the demographic characteristics, medical comorbidities, and medications of subjects with and without BPPV. Kaplan–Meier survival analysis was then performed to generate a stroke-free survival curve. Cox proportional hazard regression was performed to investigate the independent predictive value of BPPV for stroke incidence after adjusting for age, gender, hypertension, diabetes mellitus, atrial fibrillation/flutter, coronary artery disease, hyperlipidemia, antiplatelets, anticoagulants, and statins. To determine the hazard of BPPV with different confounding factors, a stratified analysis of the stroke hazard ratio of BPPV was performed. Statistical significance was defined as a two-sided *p* value ≤0.05. Furthermore, patients more than 65 years old in our study were extracted and analyzed with chi-square tests to compare the medical comorbidities between those with and without BPPV. Cox regression analysis was applied to evaluate the risk factors of ischemic stroke in patients over 65 years of age.

## Results

The average ages of the patients in the study and comparison cohorts were 57.36 ± 15.44 and 56.88 ± 15.55, respectively (*p* = 0.113). Because age and gender were matched between cohorts, no significant differences in age and gender were observed. Patients with BPPV were more likely to be accompanied by hypertension, diabetes mellitus, atrial fibrillation/flutter, coronary artery disease, and hyperlipidemia; thus, these individuals had a higher use of antiplatelets, anticoagulants, and statins. The distributions of demographic characteristics, medical comorbidities, and medication use between patients with and without BPPV are shown in Table 1.

**Table 1:** **Demographic and clinical characteristics comparisons between subjects with and without BPPV (*n* = 12483)**.

Variables	Without BPPV (*n* = 8379)		With BPPV (*n* = 4104)		*p* Value
	Total *N*	Column%	Total *N*	Column%	
Age, years (mean ± SD)	56.88 ± 15.55		57.36 ± 15.44		0.113
**GENDER**					
Female	5597	66.80	2740	66.80	0.984
Male	2782	33.20	1364	33.20	
**HTN**					
No	6134	73.20	2455	59.80	<0.001*
Yes	2245	26.80	1649	40.20	
**DM**					
No	7430	88.70	3460	84.30	<0.001*
Yes	949	11.30	644	15.70	
**AF/AFL**					
No	8305	99.10	4049	98.70	0.022*
Yes	74	0.90	55	1.30	
**CAD**					
No	7542	90.00	3322	80.90	<0.001*
Yes	837	10.00	782	19.10	
**HYPERLIPIDEMIA**					
No	7348	87.70	3263	79.50	<0.001*
Yes	1031	12.30	841	20.50	
**ANTIPLATELET**					
No	6287	75.03	2427	59.14	<0.001*
Yes	2092	24.97	1677	40.86	
**ANTICOAGULANTS**					
No	8327	99.40	4084	99.50	0.38
Yes	52	0.60	20	0.50	
**STATIN**					
No	7674	91.60	3532	86.10	<0.001*
Yes	705	8.40	572	13.90	

*p < 0.05.

*HTN, hypertension; DM, diabetes mellitus; Af/AFL, atrial fibrillation/flutter; CAD, coronary artery disease*.

**Figure 1:**
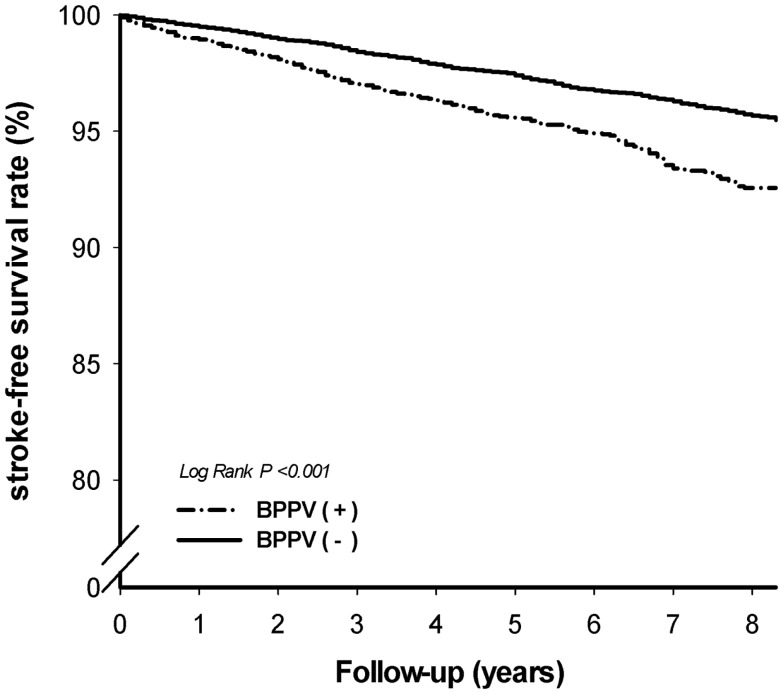
**The stroke-free survival curve was generated using Kaplan–Meier survival analysis**.

A follow-up of each of the 12,483 patients was performed until the end of 2009 to track the incidence of ischemic stroke. Of the 12,483 patients tracked, a total of 425 patients developed stroke. Of the 4104 patients with BPPV, 185 (4.5%) developed stroke, and of the 8379 patients without BPPV, 240 patients (2.9%) developed stroke. The patients with BPPV were more likely to develop ischemic stroke than those without BPPV. For BPPV patients, the crude stroke hazard ratio for developing ischemic stroke was 1.708, with a confidence interval between 1.409 and 2.069. The stroke-free survival curve was generated using Kaplan–Meier survival analysis (Figure 1). After adjustment for age, gender, hypertension, diabetes mellitus, atrial fibrillation/flutter, hyperlipidemia, coronary artery disease, and medications (including antiplatelets, anticoagulants, and statins), the hazard ratio for developing ischemic strokes during the maximal 9-year follow-up period was 1.415-fold higher in patients with BPPV than those without BPPV (confidence interval: 1.162–1.723, *p* = 0.001). Stroke incidence and the hazard ratios of BPPV are shown in Table 2.

**Table 2:** **The incidence and hazard ratio of stroke in subjects with and without BPPV**.

Presence of ischemic stroke in maximal 9-year follow-up period	Total subjects		Without BPPV		With BPPV	
	Number	%	Number	%	Number	%
Yes	425	3.4	240	2.9	185	4.5
No	12058	96.6	8139	97.1	3919	95.5
Crude hazard ratio (95% CI)			1		1.708 (1.409 ~ 2.069)**
Adjusted hazard ratio (95% CI)			1		1.415 (1.162 ~ 1.723)*

**p < 0.001, *p = 0.001.

*Adjustment was made for subjects’ age, gender, hypertension, diabetes, atrial fibrillation/flutter, hyperlipidemia, coronary artery disease, and medications including antiplatelets, coumadin, and statin*.

**Figure 2:**
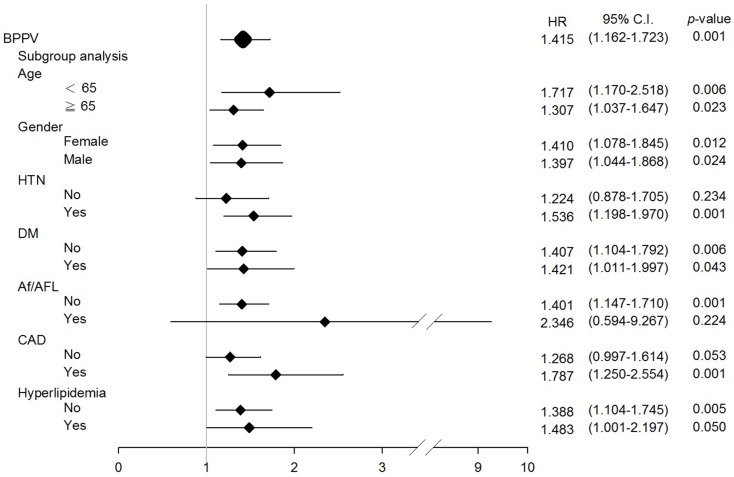
**Hazard ratios (HRs) for BPPV of ischemic stroke development in subgroup analysis**.

To further estimate the risk of developing ischemic stroke among individuals with BPPV with different comorbidities, a subgroup analysis stratified according to age, gender, hypertension, diabetes mellitus, atrial fibrillation/flutter, coronary artery disease, and hyperlipidemia was performed. BPPV remained independently associated with a higher risk of developing future ischemic stroke, regardless of age, gender, or the presence of diabetes mellitus and hyperlipidemia (Figure 2). The hazard ratio for individuals under and over 65 years of age were 1.717 and 1.307, respectively; for females and males were 1.410 and 1.397, respectively.

To determine the risk factors of developing ischemic stroke specifically among the elderly people, individuals aged over 65 years were extracted from the study and comparison cohorts (*n* = 4389). The clinical characteristics of this particular group are shown in Table 3. Elderly patients with BPPV were more likely to be comorbid with hypertension, diabetes mellitus, coronary artery disease, and hyperlipidemia. Cox regression analysis revealed that age, male gender, hypertension, diabetes mellitus, and BPPV were independently associated with increased risk of ischemic stroke (Table 4). The adjusted hazard ratio of individuals with and without BPPV is 1.307, indicating that among the elderly population, BPPV patients is at a 1.307-fold higher risk for developing subsequent ischemic stroke.

**Table 3:** **Clinical characteristics of individuals more than 65-year-old with and without BPPV (*n* = 4389)**.

Variables	Without BPPV (*n* = 2948)		With BPPV (*n* = 1441)		
	Total *N*	Column%	Total *N*	Column%	*p* Value
**GENDER**					
Female	1784	60.50	870	60.40	0.948
Male	1164	39.50	571	39.60	
**HTN**					
No	1524	51.70	526	36.50	<0.001*
Yes	1424	48.30	915	63.50	
**DM**					
No	2396	81.30	1089	75.60	<0.001*
Yes	552	18.70	352	24.40	
**AF/AFL**					
No	2881	97.70	1396	96.90	0.103
Yes	67	2.30	45	3.10	
**CAD**					
No	2349	79.70	947	65.70	<0.001*
Yes	599	20.30	494	34.30	
**HYPERLIPIDEMIA**					
No	2389	81.00	1065	73.90	<0.001*
Yes	559	19.00	376	26.10	

*p < 0.05.

*HTN, hypertension; DM, diabetes mellitus; Af/AFL, atrial fibrillation/flutter; CAD, coronary artery disease*.

**Table 4:** **Predictors of ischemic stroke in individuals more than 65 years old analyzed by Cox regression analysis**.

Predictor	Hazard ratio	95% Confidence interval	*p* Value
BPPV	1.307	1.037–1.647	0.023*
Age	1.049	1.030–1.068	<0.001*
Male gender	1.384	1.107–1.731	0.004*
HTN	1.318	1.027–1.691	0.03*
DM	1.788	1.383–2.313	<0.001*
Af/AFL	0.96	0.490–1.879	0.905
CAD	1.182	0.915–1.526	0.2
Hyperlipidemia	1.043	0.783–1.389	0.774

*p < 0.05.

*HTN, hypertension; DM, diabetes mellitus; Af/AFL, atrial fibrillation/flutter; CAD, coronary artery disease*.

## Discussion

The main finding of our study was that BPPV was associated with an increased risk of future ischemic stroke. The association between BPPV and risk of ischemic stroke remained when considering underling comorbidities, including diabetes mellitus, hypertension, coronary artery disease, and hyperlipidemia. In our study population, men over the age of 65 years were more susceptible to ischemic stroke than women, and BPPV patients in both genders were associated with increased risk of subsequent ischemic stroke.

As noted previously, BPPV results from imbalanced semicircular canal nerve stimulation by calcium deposits (Schuknecht, 1969; Hall et al., 1979). Therefore, the causes of BPPV are primarily believed to be mechanical. Because BPPV accounts for 20–30% of diagnoses in dizziness clinics (Baloh et al., 1989; Neuhauser et al., 2001) and the BPPV recurrence rate is approximately 12–20% (Steenerson et al., 2005; Kao et al., 2009), conditions that are comorbid with BPPV have been rigorously investigated in the literature. Sunami et al. (2005) reported that BPPV patients with hypertension and hyperlipidemia are more likely to experience recurrence. Using a population-based multivariate analysis, von Brevern’s cross-sectional study showed that age, migraine, hypertension, hyperlipidemia, and stroke were independently associated with BPPV (von Brevern et al., 2007). These findings are in accordance with our results, which indicated that the prevalence of hypertension, diabetes mellitus, atrial fibrillation, coronary artery disease, and hyperlipidemia is higher among patients with BPPV than among those without BPPV, leading to more frequent prescriptions for antiplatelets and statins in BPPV patients (Table 1).

Age or gender-related factors are the most likely causes of the linkage between BPPV and ischemic stroke. Cardiovascular risks increase with age, as do dizziness and vertigo. Dizziness has been reported in approximately 30% of individuals over 60 years old (Sloane et al., 1989) and the recurrence rate of BPPV was 1.7-fold higher in older individuals (Kao et al., 2009). In order to determine the effects of age and BPPV in future ischemic stroke, we analyzed the hazard ratio of stroke in the older (≥65 years old) and younger (<65 years old) populations from our database. We found that BPPV remained to be associated with subsequent ischemic strokes in both young and old groups (hazard ratio 1.717 and 1.307, respectively; Figure 2). Despite the fact that BPPV incidence increases with age, the hazard ratio is higher among the young population in our study, indicating BPPV is indeed related to future ischemic strokes regardless of age. It is known that men have a higher incidence of hemorrhagic and ischemic stroke with the exception between 35 and 44 years of age, and over 85 years (Goldstein et al., 2011). In our study population, for those who are ≥65 years of age, male gender became an independent predictor for ischemic stroke (hazard ratio = 1.384). Nevertheless, in our population, BPPV was found to be independently associated with ischemic stroke in both genders (hazard ratio in female = 1.410, in male = 1.307; Figure 2). Age and gender-related factors cannot explain the association of BPPV and formation of ischemic strokes or other cardiovascular events.

The hypothesis that decreased anterior vestibular artery blood supply causes BPPV may provide one explanation (Baloh et al., 1987). Circulation to the inner ear is from the vertebrobasilar system, primarily the anterior inferior cerebellar artery, which branches into the anterior vestibular artery. Its anatomical location within the inner ear makes the labyrinth particularly vulnerable to ischemia. Wada et al. used carotid ultrasonography to evaluate the intima–media thickness of the common carotid artery in patients with BPPV and vestibular hypofunction. This study showed that the percentage of abnormal intima–media thickness of common carotid arteries was significantly higher in patients with BPPV than those with other vestibular disorders (Wada et al., 2008). In patients with an intima–media thickness greater than 1.1 mm, the level of residual senses of positional vertigo was higher (Wada et al., 2009). A study by Zhang et al. (2013) also revealed increased abnormalities in the vertebrobasilar arteries of BPPV patients. The severity of vertigo was correlated with vertebral artery stenosis, occlusion, or tortuosity (Zhang et al., 2013). These reports are in accordance with our findings (Table 1), which showed that patients with BPPV had a higher prevalence of hypertension and coronary artery disease. Blood vessel abnormalities in the BPPV population, as demonstrated by clinical manifestations and imaging studies, could explain our findings that BPPV patients may be more susceptible to cardiovascular conditions.

Our retrospective cohort study focused on the subsequent risk of developing strokes. As shown in Table 2, an increased risk of stroke (hazard ratio = 1.415) in patients with BPPV was observed. The higher hazard ratio was still observed even the adjustment for age, gender, hypertension, diabetes mellitus, atrial fibrillation/flutter, hyperlipidemia, coronary artery disease, and medications (including antiplatelets, anticoagulants, and statins). The diagnosis of BPPV should exclude central nervous system vertigo (Fife et al., 2008). However, new hypotheses are emerging regarding whether BPPV is truly benign. One previous retrospective observational study showed that BPPV patients have increased vascular risk factors than patients with Ménière’s disease (De Reuck, 2010). This study indicated that BPPV could progress to general atherosclerosis. The study by Wada et al. suggested that patients with vestibular hypofunction may present with slow arteriosclerotic changes, likely more frequently in individuals with BPPV. In our study, we detected a higher prevalence of DM in the BPPV group. This finding is in agreement with studies by Cohen et al. (2004) and Yoda et al. (2011). One recent multicenter observational study found a higher recurrence of BPPV in patients presenting with hypertension and diabetes mellitus (De Stefano et al., 2014). Vestibular system vascular damage presenting with hypertension and diabetes mellitus could lead to higher risks of future ischemic stroke.

In our study, the subgroup analysis showed a generalized increased hazard ratio for stroke in BPPV patients with different comorbidities. These results are in accordance with one previous observation that vertigo patients with multiple risk factors had a higher risk of developing stroke (Lee et al., 2011). Our study demonstrated a specific association between BPPV and ischemic strokes. Considering underlying atrial fibrillation, an association between BPPV and ischemic stroke was not observed in our study population. There are two possible explanations for this observation. First, only 129 subjects with atrial fibrillation were included in our study; thus, a small sample size may not contain sufficient information for analysis. A larger sample size including more individuals with a diagnosis of atrial fibrillation is necessary to investigate the association of BPPV and ischemic stroke in patients with atrial fibrillation. Another explanation is that atrial fibrillation itself is the most important risk factor for stroke; therefore, BPPV may not have played a significant role leading to ischemic stroke in our patients with atrial fibrillation.

One main value of our study is the utilization of population-based data. These data allowed us to track the development of stroke in both cohorts without dropout or loss to follow-up. Furthermore, the large sample size provided sufficient power for hazard ratio analyses. However, there are limitations to our study. First, the diagnosis of stroke and BPPV primarily relied on the ICD coding recorded in the NHIRD, which may unavoidably lead to misdiagnoses. To reduce the bias caused by misdiagnoses, we only included individuals who were diagnosed with BPPV at least twice in outpatient clinics or had BPPV as the primary diagnosis as an inpatient. Regarding the diagnosis of stroke, we only included individuals who were diagnosed with stroke as inpatients because only those with a diagnosis of stroke can be admitted to hospitals in Taiwan for intensive care. The same inclusion criteria have been used in previous retrospective cohort database studies and were shown to be reliable (Chiang et al., 2011). Second, some important risk factors, such as tobacco smoking, obesity, dietary habits, and a sedentary lifestyle, were not available in our database. Investigating these risk factors is important in future studies. Finally, our database consists primarily of Taiwanese individuals, and our results may not be applicable to other ethnic groups. However, because this is the first large retrospective cohort study with East Asian individuals, it provides important information for East Asian healthcare workers. Once BPPV patients are identified, more aggressive control of modifiable risk factors, such as blood pressure, blood sugar, smoking, diet, and exercise, should be suggested to protect BPPV patients from developing stroke (Goldstein et al., 2011).

## Conclusion

Patients with BPPV were more susceptible to future ischemic strokes. Although the mechanisms of this association have yet to be identified, clinicians and BPPV patients should be aware of the likelihood of developing disabling or even life-threatening vascular events in the future. Large-scale prospective studies are necessary to provide additional insights into the etiologies of these two diseases.

## Conflict of Interest Statement

The authors declare that the research was conducted in the absence of any commercial or financial relationships that could be construed as a potential conflict of interest.
